# Supplementary material for: Pyruvate Dehydrogenase Kinase 1 inhibition mediated oxidative phosphorylation enhancement in cartilage promotes osteoarthritis progression
Source: BMC Musculoskelet Disord. 2023 Jul 20;24:597. doi: 10.1186/s12891-023-06585-6 (PMC10357736; doi:10.1186/s12891-023-06585-6)
Supplement: Supplementary file 1 — Additional file 1. [file 12891_2023_6585_MOESM1_ESM.zip › BP.Gsea.1653623667859/GOBP_GLYCOSYL_COMPOUND_METABOLIC_PROCESS.html]

Details for gene set GOBP\_GLYCOSYL\_COMPOUND\_METABOLIC\_PROCESS[GSEA]

|  || Dataset | 55457\_collapsed\_to\_symbols.cls.cls #OA\_versus\_normal.cls.cls #OA\_versus\_normal\_repos |
| Phenotype | cls.cls#OA\_versus\_normal\_repos |
| Upregulated in class | OA |
| GeneSet | GOBP\_GLYCOSYL\_COMPOUND\_METABOLIC\_PROCESS |
| Enrichment Score (ES) | 0.4391064 |
| Normalized Enrichment Score (NES) | 1.8877027 |
| Nominal p-value | 0.0 |
| FDR q-value | 0.04629488 |
| FWER p-Value | 0.796 |
Table: GSEA Results Summary

  

Fig 1: Enrichment plot: GOBP\_GLYCOSYL\_COMPOUND\_METABOLIC\_PROCESS      
 Profile of the Running ES Score & Positions of GeneSet Members on the Rank Ordered List

  

| SYMBOL | TITLE | RANK IN GENE LIST | RANK METRIC SCORE | RUNNING ES | CORE ENRICHMENT || 1 | DGUOK | deoxyguanosine kinase [Source:HGNC Symbol;Acc:HGNC:2858] | 96 | 0.917 | 0.0317 | Yes |
| 2 | ADK | adenosine kinase [Source:HGNC Symbol;Acc:HGNC:257] | 127 | 0.878 | 0.0669 | Yes |
| 3 | NME5 | NME/NM23 family member 5 [Source:HGNC Symbol;Acc:HGNC:7853] | 362 | 0.720 | 0.0794 | Yes |
| 4 | ATIC | 5-aminoimidazole-4-carboxamide ribonucleotide formyltransferase/IMP cyclohydrolase [Source:HGNC Symbol;Acc:HGNC:794] | 401 | 0.706 | 0.1067 | Yes |
| 5 | ABHD10 | "abhydrolase domain containing 10, depalmitoylase [Source:HGNC Symbol;Acc:HGNC:25656]" | 437 | 0.690 | 0.1334 | Yes |
| 6 | TYW1 | tRNA-yW synthesizing protein 1 homolog [Source:HGNC Symbol;Acc:HGNC:25598] | 523 | 0.650 | 0.1546 | Yes |
| 7 | NME4 | NME/NM23 nucleoside diphosphate kinase 4 [Source:HGNC Symbol;Acc:HGNC:7852] | 677 | 0.599 | 0.1682 | Yes |
| 8 | DNPH1 | 2'-deoxynucleoside 5'-phosphate N-hydrolase 1 [Source:HGNC Symbol;Acc:HGNC:21218] | 719 | 0.589 | 0.1902 | Yes |
| 9 | AKR1A1 | aldo-keto reductase family 1 member A1 [Source:HGNC Symbol;Acc:HGNC:380] | 808 | 0.567 | 0.2076 | Yes |
| 10 | ENPP4 | ectonucleotide pyrophosphatase/phosphodiesterase 4 [Source:HGNC Symbol;Acc:HGNC:3359] | 822 | 0.563 | 0.2306 | Yes |
| 11 | GUSB | glucuronidase beta [Source:HGNC Symbol;Acc:HGNC:4696] | 904 | 0.541 | 0.2475 | Yes |
| 12 | LCMT2 | leucine carboxyl methyltransferase 2 [Source:HGNC Symbol;Acc:HGNC:17558] | 946 | 0.529 | 0.2669 | Yes |
| 13 | DERA | deoxyribose-phosphate aldolase [Source:HGNC Symbol;Acc:HGNC:24269] | 975 | 0.523 | 0.2870 | Yes |
| 14 | ICMT | isoprenylcysteine carboxyl methyltransferase [Source:HGNC Symbol;Acc:HGNC:5350] | 1140 | 0.488 | 0.2951 | Yes |
| 15 | APRT | adenine phosphoribosyltransferase [Source:HGNC Symbol;Acc:HGNC:626] | 1178 | 0.481 | 0.3128 | Yes |
| 16 | APOBEC3C | apolipoprotein B mRNA editing enzyme catalytic subunit 3C [Source:HGNC Symbol;Acc:HGNC:17353] | 1355 | 0.450 | 0.3183 | Yes |
| 17 | NUDT1 | nudix hydrolase 1 [Source:HGNC Symbol;Acc:HGNC:8048] | 1425 | 0.439 | 0.3317 | Yes |
| 18 | FUCA1 | alpha-L-fucosidase 1 [Source:HGNC Symbol;Acc:HGNC:4006] | 1506 | 0.428 | 0.3437 | Yes |
| 19 | NAGA | alpha-N-acetylgalactosaminidase [Source:HGNC Symbol;Acc:HGNC:7631] | 1674 | 0.402 | 0.3479 | Yes |
| 20 | GBA3 | glucosylceramidase beta 3 (gene/pseudogene) [Source:HGNC Symbol;Acc:HGNC:19069] | 1767 | 0.388 | 0.3573 | Yes |
| 21 | AICDA | activation induced cytidine deaminase [Source:HGNC Symbol;Acc:HGNC:13203] | 1825 | 0.381 | 0.3692 | Yes |
| 22 | PTGDR | prostaglandin D2 receptor [Source:HGNC Symbol;Acc:HGNC:9591] | 1904 | 0.372 | 0.3790 | Yes |
| 23 | APOBEC3G | apolipoprotein B mRNA editing enzyme catalytic subunit 3G [Source:HGNC Symbol;Acc:HGNC:17357] | 1917 | 0.370 | 0.3939 | Yes |
| 24 | ADA2 | adenosine deaminase 2 [Source:HGNC Symbol;Acc:HGNC:1839] | 1955 | 0.367 | 0.4067 | Yes |
| 25 | AKR1C3 | aldo-keto reductase family 1 member C3 [Source:HGNC Symbol;Acc:HGNC:386] | 1996 | 0.361 | 0.4190 | Yes |
| 26 | CBR4 | carbonyl reductase 4 [Source:HGNC Symbol;Acc:HGNC:25891] | 2565 | 0.295 | 0.3874 | Yes |
| 27 | AKR7A2 | aldo-keto reductase family 7 member A2 [Source:HGNC Symbol;Acc:HGNC:389] | 2690 | 0.280 | 0.3897 | Yes |
| 28 | APOBEC1 | apolipoprotein B mRNA editing enzyme catalytic subunit 1 [Source:HGNC Symbol;Acc:HGNC:604] | 2695 | 0.280 | 0.4013 | Yes |
| 29 | OARD1 | O-acyl-ADP-ribose deacylase 1 [Source:HGNC Symbol;Acc:HGNC:21257] | 2713 | 0.278 | 0.4119 | Yes |
| 30 | PRPSAP2 | phosphoribosyl pyrophosphate synthetase associated protein 2 [Source:HGNC Symbol;Acc:HGNC:9467] | 2855 | 0.265 | 0.4122 | Yes |
| 31 | TK1 | thymidine kinase 1 [Source:HGNC Symbol;Acc:HGNC:11830] | 2909 | 0.259 | 0.4192 | Yes |
| 32 | UMPS | uridine monophosphate synthetase [Source:HGNC Symbol;Acc:HGNC:12563] | 2936 | 0.257 | 0.4281 | Yes |
| 33 | AKR1B1 | aldo-keto reductase family 1 member B [Source:HGNC Symbol;Acc:HGNC:381] | 3138 | 0.236 | 0.4225 | Yes |
| 34 | MTAP | methylthioadenosine phosphorylase [Source:HGNC Symbol;Acc:HGNC:7413] | 3324 | 0.219 | 0.4175 | Yes |
| 35 | GBA | glucosylceramidase beta [Source:HGNC Symbol;Acc:HGNC:4177] | 3327 | 0.219 | 0.4267 | Yes |
| 36 | NT5E | 5'-nucleotidase ecto [Source:HGNC Symbol;Acc:HGNC:8021] | 3515 | 0.201 | 0.4207 | Yes |
| 37 | DTYMK | deoxythymidylate kinase [Source:HGNC Symbol;Acc:HGNC:3061] | 3538 | 0.199 | 0.4274 | Yes |
| 38 | TYMP | thymidine phosphorylase [Source:HGNC Symbol;Acc:HGNC:3148] | 3588 | 0.195 | 0.4320 | Yes |
| 39 | APOBEC3F | apolipoprotein B mRNA editing enzyme catalytic subunit 3F [Source:HGNC Symbol;Acc:HGNC:17356] | 3603 | 0.193 | 0.4391 | Yes |
| 40 | PRPS1 | phosphoribosyl pyrophosphate synthetase 1 [Source:HGNC Symbol;Acc:HGNC:9462] | 3717 | 0.184 | 0.4381 | No |
| 41 | DPYD | dihydropyrimidine dehydrogenase [Source:HGNC Symbol;Acc:HGNC:3012] | 3830 | 0.173 | 0.4368 | No |
| 42 | GLA | galactosidase alpha [Source:HGNC Symbol;Acc:HGNC:4296] | 4172 | 0.140 | 0.4162 | No |
| 43 | APOBEC2 | apolipoprotein B mRNA editing enzyme catalytic subunit 2 [Source:HGNC Symbol;Acc:HGNC:605] | 4279 | 0.130 | 0.4135 | No |
| 44 | PRPS2 | phosphoribosyl pyrophosphate synthetase 2 [Source:HGNC Symbol;Acc:HGNC:9465] | 4365 | 0.123 | 0.4122 | No |
| 45 | MACROD1 | mono-ADP ribosylhydrolase 1 [Source:HGNC Symbol;Acc:HGNC:29598] | 5008 | 0.072 | 0.3652 | No |
| 46 | APOBEC3B | apolipoprotein B mRNA editing enzyme catalytic subunit 3B [Source:HGNC Symbol;Acc:HGNC:17352] | 5089 | 0.065 | 0.3618 | No |
| 47 | ERH | ERH mRNA splicing and mitosis factor [Source:HGNC Symbol;Acc:HGNC:3447] | 5109 | 0.063 | 0.3630 | No |
| 48 | TH | tyrosine hydroxylase [Source:HGNC Symbol;Acc:HGNC:11782] | 5374 | 0.042 | 0.3442 | No |
| 49 | AKR1C4 | aldo-keto reductase family 1 member C4 [Source:HGNC Symbol;Acc:HGNC:387] | 5843 | 0.005 | 0.3080 | No |
| 50 | ACP3 | acid phosphatase 3 [Source:HGNC Symbol;Acc:HGNC:125] | 6271 | -0.025 | 0.2758 | No |
| 51 | HPRT1 | hypoxanthine phosphoribosyltransferase 1 [Source:HGNC Symbol;Acc:HGNC:5157] | 6954 | -0.076 | 0.2259 | No |
| 52 | TK2 | thymidine kinase 2 [Source:HGNC Symbol;Acc:HGNC:11831] | 7468 | -0.116 | 0.1909 | No |
| 53 | ADA | adenosine deaminase [Source:HGNC Symbol;Acc:HGNC:186] | 8105 | -0.163 | 0.1483 | No |
| 54 | AKR1C1 | aldo-keto reductase family 1 member C1 [Source:HGNC Symbol;Acc:HGNC:384] | 8198 | -0.171 | 0.1484 | No |
| 55 | CDA | cytidine deaminase [Source:HGNC Symbol;Acc:HGNC:1712] | 8324 | -0.180 | 0.1464 | No |
| 56 | XDH | xanthine dehydrogenase [Source:HGNC Symbol;Acc:HGNC:12805] | 8366 | -0.183 | 0.1510 | No |
| 57 | PRPS1L1 | phosphoribosyl pyrophosphate synthetase 1 like 1 [Source:HGNC Symbol;Acc:HGNC:9463] | 8382 | -0.184 | 0.1577 | No |
| 58 | AKR1B10 | aldo-keto reductase family 1 member B10 [Source:HGNC Symbol;Acc:HGNC:382] | 8420 | -0.187 | 0.1628 | No |
| 59 | SLC34A1 | solute carrier family 34 member 1 [Source:HGNC Symbol;Acc:HGNC:11019] | 8919 | -0.226 | 0.1336 | No |
| 60 | GNMT | glycine N-methyltransferase [Source:HGNC Symbol;Acc:HGNC:4415] | 9208 | -0.248 | 0.1218 | No |
| 61 | NT5C2 | "5'-nucleotidase, cytosolic II [Source:HGNC Symbol;Acc:HGNC:8022]" | 9595 | -0.283 | 0.1038 | No |
| 62 | UPB1 | beta-ureidopropionase 1 [Source:HGNC Symbol;Acc:HGNC:16297] | 9609 | -0.284 | 0.1149 | No |
| 63 | PNP | purine nucleoside phosphorylase [Source:HGNC Symbol;Acc:HGNC:7892] | 9657 | -0.288 | 0.1236 | No |
| 64 | AMD1 | adenosylmethionine decarboxylase 1 [Source:HGNC Symbol;Acc:HGNC:457] | 10415 | -0.363 | 0.0801 | No |
| 65 | APOBEC3A | apolipoprotein B mRNA editing enzyme catalytic subunit 3A [Source:HGNC Symbol;Acc:HGNC:17343] | 10550 | -0.378 | 0.0858 | No |
| 66 | UCKL1 | uridine-cytidine kinase 1 like 1 [Source:HGNC Symbol;Acc:HGNC:15938] | 11515 | -0.503 | 0.0321 | No |
| 67 | PPAT | phosphoribosyl pyrophosphate amidotransferase [Source:HGNC Symbol;Acc:HGNC:9238] | 11534 | -0.505 | 0.0523 | No |
| 68 | UPP1 | uridine phosphorylase 1 [Source:HGNC Symbol;Acc:HGNC:12576] | 12013 | -0.595 | 0.0405 | No |
| 69 | CDADC1 | cytidine and dCMP deaminase domain containing 1 [Source:HGNC Symbol;Acc:HGNC:20299] | 12267 | -0.667 | 0.0493 | No |
Table: GSEA details [plain text format]

  

Fig 2: GOBP\_GLYCOSYL\_COMPOUND\_METABOLIC\_PROCESS      
 Blue-Pink O' Gram in the Space of the Analyzed GeneSet

  

Fig 3: GOBP\_GLYCOSYL\_COMPOUND\_METABOLIC\_PROCESS: Random ES distribution      
 Gene set null distribution of ES for **GOBP\_GLYCOSYL\_COMPOUND\_METABOLIC\_PROCESS**
